# Blood Microbial Communities During Pregnancy Are Associated With Preterm Birth

**DOI:** 10.3389/fmicb.2019.01122

**Published:** 2019-06-04

**Authors:** Young-Ah You, Jae Young Yoo, Eun Jin Kwon, Young Ju Kim

**Affiliations:** Department of Obstetrics and Gynecology, Ewha Medical Research Institute, Ewha Womans University School of Medicine, Seoul, South Korea

**Keywords:** blood microbiota, pregnancy, preterm birth, 16S rRNA gene sequencing, microbiome

## Abstract

Microbial infection of the placenta, amniotic fluid, vaginal canal, and oral cavity is known to significantly contribute to preterm birth (PTB). Although microbes can be translocated into the blood, little is known regarding the blood microbiota during pregnancy. To assess changes in the microbiome during pregnancy, blood samples were obtained 2 or 3 times during pregnancy from a cohort of 45 pregnant women enrolled between 2008 and 2010. To analyze the association with PTB, we conducted a case-control study involving 41 pregnant women upon admission for preterm labor and rupture of membrane (20 with term delivery; 21 with PTB). Bacterial diversity was assessed in number and composition between the first, second, and third trimesters in term delivered women according to 16S rRNA gene amplicon sequencing, and data were analyzed using Quantitative Insight Into Microbial Ecology (QIIME). Taxonomy was assigned using the GreenGenes 8.15.13 database. Dominant microorganisms at the phylum level in all pregnant women were identified as *Firmicutes*, *Proteobacteria*, *Bacteroidetes*, and *Actinobacteria*. However, the number and composition of bacteria in women with PTB differed from that in women with term delivery. *Firmicutes* and *Bacteroidetes* were more abundant in women with PTB than in women with term delivery, while *Proteobacteria* was less prevalent in women with PTB. At the genus level, *Bacteroides*, *Lactobacillus*, *Sphingomonas*, *Fastidiosipila*, *Weissella*, and *Butyricicoccus* were enriched in PTB samples. These observational results suggest that several taxa in the maternal blood microbiome are associated with PTB. Further studies are needed to confirm the composition of the blood microbiota in women with PTB. Additionally, the mechanism by which pathogenic microbes in maternal blood cause infection and PTB requires further analysis.

## Introduction

Approximately 15 million babies are born prematurely each year (Lawn et al., 2013), defined as parturition before 37 weeks of gestation. In Korea, the rate of preterm birth (PTB) has continuously increased by 1.4-fold, from 4.7% in 2005 to 6.9% in 2015 (Hwang et al., 2015; Korean Statistical Information Service, 2016). Prematurely born babies account for a significant proportion of infant morbidity and mortality (Simmons et al., 2010; Yoo et al., 2018). Approximately 40% of PTBs spontaneously occur because of infection and inflammation, including associations with subclinical intrauterine, intra-amniotic, and extrauterine maternal infections, such as periodontal disease (Goldenberg and Culhane, 2006; Mysorekar and Cao, 2014).

Over the course of pregnancy, the microbiome in every organ of the body undergoes profound changes associated with metabolic alterations and immunological adaptations (Koren et al., 2012). However, bacterial pathogens and associated products can induce local inflammatory responses in gestational tissues (acute chorioamnionitis), leading to preterm labor (Romero et al., 1988, 1989; Yoon et al., 1998). Most intra-amniotic infections are thought to occur when the microbiome in the lower genital tract (vagina and/or cervix) gains access to the amniotic fluid (Romero et al., 2006). Changes in the microbial ecosystem of the vagina have been implicated in the genesis of ascending intrauterine infection (Hillier et al., 1995; Hitti et al., 2001; Racicot et al., 2013). Similarly, microbial infection of the amniotic fluid, vaginal canal, and oral cavity is known to significantly contribute to PTB (Ryan and Ray, 2004; Menon, 2008; Yoo et al., 2016).

High-throughput, culture-independent technologies can be used to assess the microbiome of each organ in the body at high taxonomic resolution (Hillier et al., 1995; Hitti et al., 2001; Menon, 2008; Racicot et al., 2013). We previously reported analyses of the microbial communities of amniotic fluid and urine by sequencing of the bacterial 16S ribosomal RNA gene in women who delivered preterm (Yoo et al., 2016; You et al., 2016). We identified *Sneathia sanguinegens* and *Fusobacterium nucleatum* in amniotic fluid samples from two preterm delivered women (You et al., 2016). In urine samples, *Ureaplasma* spp. and *Veillonellaceae* family members, including *Megasphaera* spp., were more abundant in preterm delivered women than in women who delivered at term (Yoo et al., 2016). While antibiotic use can alter the composition and structure of the microbiota in specific cases, such as in chorioamnionitis and cervical infection (You et al., 2016), microbes such as *F. nucleatum*, *Leptotrichia* (*Sneathia*), *Ureaplasma urealyticum*, *Mycoplasma hominis*, *Streptococcus agalactiae*, *Escherichia coli*, and a species of the order *Clostridiales* can induce local inflammatory responses in gestational tissues, causing PTB (Peltier, 2003; Han et al., 2006; Wang et al., 2013). However, the microbial composition of the blood of women who undergo preterm delivery has not been established.

The aim of this study was to characterize the composition of the blood microbiome during healthy pregnancy and to compare the blood microbiome of pregnant women with those of term and preterm delivery using 16S rRNA gene sequencing-based methods.

## Materials and Methods

### Study Population

To analyze changes in the microbiome during pregnancy, blood samples were obtained 2 or 3 times from 45 pregnant women enrolled in a cohort study between 2008 and 2010 when they visited Ewha Womans University MokDong Hospital for regular pregnancy check-ups in the first, second, and third trimesters. Only pregnant women who underwent term delivery with healthy singletons were included in this cohort. We also conducted a case-control study involving 41 pregnant women (20 with term delivery; 21 with PTB) between 2014 and 2015 to compare the blood microbiome of pregnant women with term and preterm delivery. The normal term delivery group (who underwent term delivery at ≥37 weeks of gestation) was selected from among women who had undergone prenatal examinations and were followed up until delivery in our hospital. When pregnant women in the case-control study were admitted for the first time with symptoms of labor and/or rupture of membrane, maternal blood was collected in EDTA-containing tubes, and blood cells and plasma were separated within 24 h and stored at –70°C. The inclusion criteria were a singleton birth and gestational age of 25–42 weeks at delivery. We excluded women who had multiple births, stillbirths, infants with congenital anomalies, chronic hypertension, pregnancy complication, placenta previa, and abruption placenta.

### Ethics Statement

The present study was approved by the Institutional Review Board of Ewha Womans University Hospital (ECT 127-07 and EUMC 2014-06-010). The methods were conducted in accordance with the approved guidelines. All participants were fully informed regarding the study and provided written informed consent.

### DNA Extraction and 16S rRNA Gene Sequencing

Bacterial DNA was extracted from batches of blood cells using a PowerMax Soil DNA Isolation Kit (MOBIO, Carlsbad, CA, United States) following the manufacturer’s protocol. The V3–V4 hypervariable region of bacterial genomic DNA was amplified according to Illumina 16S metagenomic sequencing library protocols (Illumina, San Diego, CA, United States). The barcoded fusion primer sequences used for amplification were 16S_V3_F (5′-TCGTCGGCAGCGTCAGATGTGTATAAGAGACAGCCTACG GGNGGCWGCAG-3′) and 16S_V4_R (5′-GTCTCGTGGGCT CGGAGATGTGTATAAGAGACA GGACTACHVGGGTATCT AATCC-3′). The libraries were prepared using PCR products according to the MiSeq System guide (Illumina) and quantified using a QIAxpert (QIAGEN, Hilden, Germany). After PCR products were extracted and quantified, equimolar ratios from each mixture were pooled and sequenced on the MiSeq (Illumina) platform according to the manufacturer’s recommendations.

### Analysis of Blood Microbiome

Raw pyrosequencing reads obtained from the sequencer were filtered according to the barcode and primer sequences using MiSeq (Illumina). Taxonomic assignment was performed with the profiling program MDx-Pro ver.1 (MD Healthcare, Seoul, Korea). High-quality sequencing reads were selected after filtering based on read length (≥300 bp) and quality score (average Phred score ≥20). Operational taxonomic units (OTUs) were clustered using the sequence clustering algorithm CD-HIT (Fu et al., 2012). Subsequently, taxonomy assignment was performed using UCLUST (Edgar, 2010) and QIIME (Caporaso et al., 2010) against the 16S rRNA gene sequence database in GreenGenes 8.15.13^1^. All 16S rRNA gene sequences were assigned to taxonomic levels based on sequence similarity. The bacterial composition at each level was plotted as a stack bar. When case clusters could not be assigned at the genus level because of a lack of sequences or redundant sequences in the database, taxa were assigned at higher levels, which are indicated in parentheses. Data were normalized to have a mean of 0 and standard deviation of 1 by linear normalization. Principal coordinate analysis and two-dimensional scatter plots with axes of the first and second principal components were calculated and drawn using Matlab 2011a (Lee et al., 2017).

### Statistical Analysis

Results are presented as the mean ± standard deviation. Basic patient characteristics, including age, maternal features, and birth outcomes, were compared between term and preterm delivered women using a Student *t*-test. Based on significant differences in the Shannon index, clustering characteristics were compared using the Kruskal–Wallis test (PERMANOVA). Statistical analyses were performed using SAS software (Version 9.3; SAS Institute, Cary, NC, United States). Results were considered statistically significant when the probability value (*p*) was <0.05.

## Results

### Blood Microbial Diversity and Composition of Pregnant Women in Cohort

To characterize the blood microbial composition during healthy pregnancy, we obtained blood samples from 45 subjects in their first, second, and third trimesters. Mean patient ages and gestational ages at delivery were 32.0 years (range: 26–40 years) and 39 weeks 3 days (range: 37 weeks 1 day–41 weeks 2 days), respectively. The data set comprised 1,609,012 high-quality gene sequences from blood samples, with an average of 16,243 reads per sample. After filtering out low-quality reads and trimming extra-long tails, the remaining representative reads were clustered into OTUs based on a 97% sequence similarity cut-off at the genus level.

**FIGURE 1:**
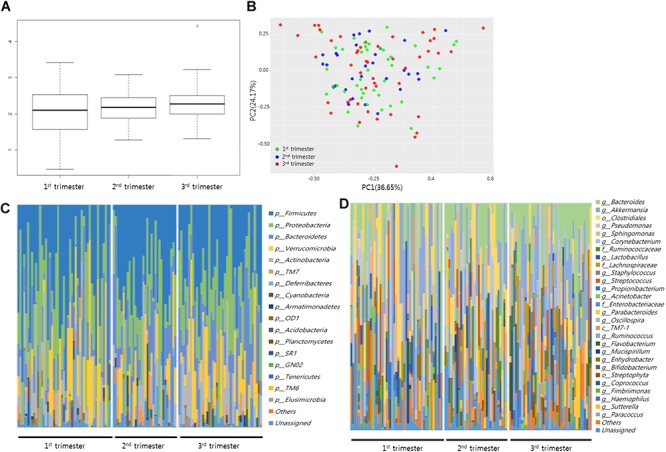
Microbial diversity and profiling during healthy pregnancy by 16S rRNA gene sequencing. **(A)** Comparison of Shannon index (community richness) in first, second, and third trimesters in maternal blood. **(B)** Plot of principal component analysis. Relative abundances of operational taxonomic units (OTUs) accounting for >0.1% of the total bacterial community are shown. **(C)** Bacterial profiling plot of relative abundances of OTUs at the phylum level and **(D)** genus level.

We analyzed the taxonomic diversity and profiles of bacterial DNA sequences in the blood during healthy pregnancy by assessing the number and abundance of distinct types of organisms. Figure 1 presents the Shannon index, principal component analysis, and relative abundances of OTUs at the phylum and genus levels in each sample. Analysis of the Shannon index and principal component analysis did not identify significant differences among the first, second, and third trimesters. Microorganisms with a relative abundance of >0.1% at the phylum level predominantly included *Firmicutes*, *Proteobacteria*, *Bacteroidetes*, and *Actinobacteria*, and those at the genus level included *Bacteroides*, *Pseudomonas*, *Sphingomonas*, *Ruminococcaceae*, *Staphylococcus*, *Propionibacterium*, and *Streptococcus*.

### Blood Microbial Diversity and Composition of Pregnant Women in Case-Control Study

To compare the bacterial compositions of term and preterm groups, we analyzed the bacterial diversity and relative abundances of OTUs in those in the case-control study. The general characteristics of the pregnant women in this study are shown in Table 1. The mean ages of the women in the term and preterm groups were 31.6 and 30.9 years, respectively. Gestational age at delivery, weight of the neonate, and Apgar score were significantly lower in the preterm group (*p* < 0.05).

**TABLE 1:** General characteristics of study subjects.

	**Women with term delivery (*N* = 20)**	**Women with preterm delivery (*N* = 21)**	***p*-value**
**Mothers**			
Age (years)	31.60 ± 2.91	30.91 ± 4.37	0.55
BMI (kg/m^2^)	26.81 ± 3.13	26.12 ± 4.67	0.59
**Parity, *n* (%)**			
Nulliparous	10 (50.0)	9 (42.9)	0.71
Multiparous	10 (50.0)	12 (57.1)	
**Mode of delivery, *n* (%)**			
Vaginal	13 (65.0)	16 (76.2)	0.43
C-section	7 (35.0)	5 (23.8)	
**Education (years)**			
≤12	5 (26.3)	10 (52.6)	0.10
>12	14 (73.7)	9 (47.4)	
**Infants**			
Gestational age (weeks)	39.65 ± 1.04^a^	29.67 ± 3.58	<0.0001
**Sex, *n* (%)**			
Male	11 (55.0)	12 (57.1)	0.89
Female	9 (45.0)	9 (42.9)	
Weight (kg)	3.36 ± 0.36^a^	1.48 ± 0.60	<0.0001
AS at 1 min	9.65 ± 0.59^a^	6.33 ± 3.06	<0.0001
AS at 5 min	10.00 ± 0.00^a^	7.48 ± 3.12	0.001

*Data are expressed as means ± SD or number (%). ^a^*p*-values were calculated by Student *t*-test. BMI, body mass index of pregnant women at delivery; C-section, cesarean section; and AS, Apgar score.*

**FIGURE 2:**
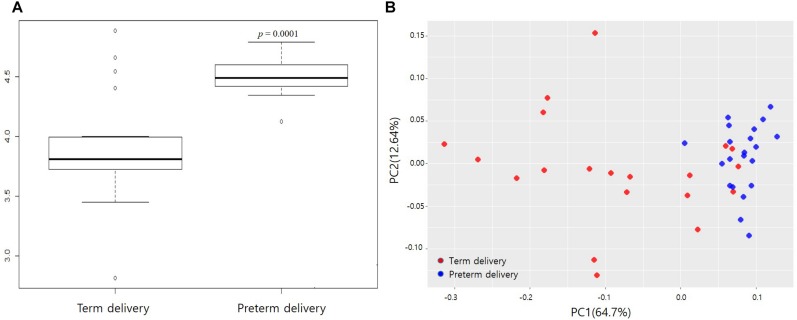
Differences in microbial diversity between term and preterm delivery. **(A)** Comparison of Shannon index (community richness) between term and preterm delivery (*p* = 2.02E-10). **(B)** Plot of principal component analysis. Relative abundances of operational taxonomic units (OTUs) accounting for >0.1% of the total bacterial community are shown.

The data set comprised 1,679,505 high-quality gene sequences, with an average of 16,243 reads per sample. After filtering out low-quality reads and trimming extra-long tails, the remaining representative reads were clustered into OTUs with a 97% sequence similarity cut-off at the genus level. The Shannon index was significantly higher in the preterm group than the term group (*p* < 0.05). The principal component analysis revealed differences between the term and preterm groups (Figure 2).

The blood microbiota of the two groups were enriched primarily in *Firmicutes*, *Bacteroidetes*, *Proteobacteria*, and *Actinobacteria*. While *Firmicutes* and *Bacteroidetes* were more abundant in women with PTB than in women with term delivery (*p* < 0.05), *Proteobacteria* was less abundant in women with PTB (Supplementary Table S1, *p* < 0.001). Notably, at the class level, *Betaproteobacteria* and *Gammaproteobacteria* were significantly less abundant in women with PTB than in those with term delivery (Supplementary Table S2, *p* < 0.001). Among genera with abundances of >0.1%, *Bacteroides*, *Lactobacillus*, *Sphingomonas*, *Fastidiosipila*, and *Butyricicoccus* were enriched in preterm samples, after adjusting for maternal age, pregnant BMI, delivery mode, and sex of the newborn (Table 2). At the genus level, the archaeon *Methanobrevibacter* and uncultured bacteria belonging to *Ruminococcaceae*, *Saccharibacteria*, and *Lachnospiraceae* were enriched in the preterm samples. In contrast, *Delftia*, *Pseudomonas*, *Massilia*, and *Stenotrophomonas* belonging to the *Proteobacteria* phylum were enriched in term samples (*p* < 0.05).

**TABLE 2:** Abundances of genera differed in peripheral blood of women who experienced preterm and term delivery.

**Taxon**	**Term delivery**		**Preterm delivery**		*p*-value^*^	**Fold**
	**Mean**	**SD**	**Mean**	**SD**		**change**
Bacteroides	3.425	2.614	8.722	1.891	9.01E-09	2.55
Lactobacillus	1.665	1.175	3.391	1.353	1.30E-04	2.04
Sphingomonas	0.944	0.938	2.051	0.911	5.91E-04	2.17
Rhizobium	1.192	0.661	1.898	1.046	1.65E-02	1.59
Clostridiales vadinBB60 group	0.758	0.767	1.353	0.693	1.52E-02	1.78
Ruminococcaceae	0.207	0.407	0.852	0.440	2.86E-05	4.11
Delftia	3.050	2.263	0.828	0.359	4.14E-04	0.27
Eisenbergiella	0.474	0.554	0.814	0.428	3.78E-02	1.72
Pseudomonas	3.680	2.913	0.800	0.502	3.89E-04	0.22
Ruminiclostridium 5	0.388	0.596	0.754	0.398	2.87E-02	1.95
Fastidiosipila	0.130	0.268	0.603	0.414	1.42E-04	4.65
Saccharibacteria	0.134	0.264	0.466	0.344	1.71E-03	3.47
Lachnospiraceae	0.163	0.315	0.449	0.414	2.01E-02	2.76
Syntrophaceticus	0.120	0.230	0.373	0.307	6.25E-03	3.10
Butyricicoccus	0.045	0.135	0.277	0.234	5.84E-04	6.14
Massilia	0.743	0.701	0.272	0.219	1.03E-02	0.37
DA101 soil group	0.096	0.172	0.256	0.198	1.09E-02	2.65
Stenotrophomonas	1.395	1.313	0.230	0.232	1.09E-03	0.17
Anaerolineaceae	0.058	0.145	0.221	0.207	6.91E-03	3.83
Methanobrevibacter	0.015	0.066	0.186	0.336	3.59E-02	12.40
Methanocella	0.023	0.039	0.171	0.280	2.91E-02	7.56
Flavonifractor	0.024	0.072	0.170	0.166	1.23E-03	7.10
Woesearchaeota (DHVEG-6)	0.026	0.113	0.150	0.205	2.51E-02	5.69
Prochlorococcus	0.017	0.053	0.137	0.198	1.54E-02	7.90
Gemmata	0.024	0.062	0.122	0.185	3.30E-02	5.19
Morus notabilis	0.027	0.090	0.122	0.149	2.08E-02	4.53
planctomycete WY108	0.017	0.072	0.117	0.167	2.02E-02	6.94
[Eubacterium] hallii group	0.010	0.038	0.108	0.143	7.06E-03	11.17

^*^p-values were calculated using ANCOVA adjusting for maternal age, pregnant BMI, type of delivery mode, and newborn’s gender.

## Discussion

Our study investigated the characteristics of the blood microbiota during healthy pregnancy and its association with PTB by sequencing the V3–V4 region of the 16S rRNA gene. During healthy pregnancy, bacterial diversity was similar in number and composition between the first, second, and third trimesters. However, the microbial diversity in women with PTB differed from that in women with term delivery. The blood microbiome of all pregnant women enrolled in the study was largely composed of nonpathogenic commensal bacteria from the *Firmicutes*, *Bacteroidetes*, *Proteobacteria*, and *Actinobacteria* phyla. While bacterial enrichment during healthy pregnancy did not differ from this overall picture, several taxa, such as *Bacteroides*, *Lactobacillus*, *Delftia*, and *Pseudomonas* exhibited differential enrichment between blood samples of women who delivered preterm and term in a case-control study. Our results suggest that several taxa in the maternal blood microbiome are associated with PTB.

In healthy pregnancy, the microbiota in all organs of the body undergoes profound changes associated with metabolic alterations and immunological adaptations (Goldenberg and Culhane, 2006). Moreover, the similarity between the oral and placental microbiome suggests that the placental microbiome becomes colonized primarily as the result of hematogenous bacterial spread via the circulation (Ramos et al., 2015). Bacterial infections threaten pregnant women and the fetus by gaining access to gestational tissues, such as the decidua, placenta, and fetal membranes (Vinturache et al., 2016). Notably, the virulence properties assigned to specific oral pathogenic bacteria, for example, *F. nucleatum*, *Porphyromonas gingivalis*, *Filifactor alocis*, *Campylobacter rectus*, and others, render them potential collaborators in adverse outcomes of pregnancy (Cobb et al., 2017), and these pathogenic bacteria can be transmitted from the oral cavity to gestational tissues via hematogenous spread (Han et al., 2006; Aagaard et al., 2014).

Based on our results, the richness of the microbial community (Shannon index) did not differ among trimesters during healthy pregnancy, but it was increased in the blood samples of women with preterm delivery compared to that of term delivery. This indicates that the community structure of the blood microbiota in women with PTB differs from that in women with term delivery, even though the microbial community structure does not change during healthy pregnancy. However, a previous study reported that PTB is associated with distinct microbial DNA changes detected in midtrimester maternal serum (Subramaniam et al., 2018). Another study reported the increased richness and diversity of the vaginal microbiome in spontaneous PTB (Freitas et al., 2018). These findings suggest that a more diverse microbiome may be important in the pathogenesis of some bacteria.

The microbial communities of all pregnant women in the study were primarily composed of *Firmicutes*, *Bacteroidetes*, *Proteobacteria*, and *Actinobacteria*. This composition of the blood microbiota is similar to that of the human gut microbiota (Khanna and Tosh, 2014). In our study, while the composition did not change during healthy pregnancy, *Firmicutes* and *Bacteroidetes* were enriched in the samples of women delivered preterm, while *Proteobacteria* was reduced. A previous study reported that the gut microbiota consisted of mostly *Firmicutes* and *Bacteroidetes* in the first trimester but shifted substantially in phylogenetic composition and structure over the course of pregnancy (Goldenberg and Culhane, 2006). The enrichment of *Proteobacteria* and *Actinobacteria* was observed during the third trimester of pregnancy in most cases (Goldenberg and Culhane, 2006). Notably, enrichment of the *Proteobacteria* in the third trimester has been observed repeatedly under inflammation-associated dysbiosis (Païssé et al., 2016). Although pregnancy is not a disease, this suggests that a shift in the gut microbiota during pregnancy causes dysbiosis of the blood microbiota.

The class *Bacteroidia* of the phylum *Bacteroidetes* is composed of a single order of environmental bacteria. *Bacteroides* spp. provide some benefits to their host by excluding potential pathogens (Stevens et al., 2012). However, *Bacteroides* such as *B. fragilis* and *B. thetaiotaomicron* can quickly become opportunistic pathogens if they are translocated outside the gastrointestinal tract, and they have been associated with abscess formation across multiple body sites, such as the abdomen, brain, liver, pelvis, and lungs, as well as serious bloodstream infections (McGregor et al., 1991; Wexler, 2007). Phospholipase C production by *B. fragilis*, *B. bivius*, and *B. thetaiotaomicron* has been implicated in various reproductive tract infections as well as PTB (McGregor et al., 1991). In addition, among women in preterm labor, a study reported an increased rate of preterm delivery (≤34 weeks) in pregnant women with high concentrations of *B. bivius* and *B. fragilis* in their vaginal fluid (Krohn et al., 1991).

Abundant *Lactobacillus* spp. in the vaginal microbiome are important for maintaining pregnancy (Romero et al., 2014). However, our results showed that *Lactobacillus* was more abundant in the blood of women with PTB compared to that in women with term delivery. A study reported that enrichment of *Lactobacillales* was frequently observed in the intestinal microbiota of women in a PTB group compared to that in a term delivered group (Sato et al., 2014; Shiozaki et al., 2014). Although *Pseudomonas* commonly causes conjunctivitis in hospitalized preterm infants (Shah et al., 1999), *Pseudomonas*, belonging to the *Proteobacteria*, was enriched in term samples in this study. In addition, *Delftia*, which in amniotic fluid has been linked to PTB (DiGiulio et al., 2008), was also enriched in term samples. Further study is needed to explain the relatively high abundance of *Lactobacillus* and the deficiency of *Pseudomonas* and *Delftia* in preterm blood samples compared with that in term samples.

Although the *Firmicutes* to *Bacteroidetes* ratio in stool samples is associated with obesity (Ley et al., 2005; Schwiertz et al., 2010), *Bacteroides* and *Lactobacillus* were still enriched in preterm samples in this study. In addition, maternal age, delivery mode, and newborn sex did not affect the enrichment of *Bacteroides* and *Lactobacillus* in preterm samples. A previous study reported that infant sex contributes to the dynamic development of the gut microbiome in preterm infants (Cong et al., 2016). The study reported that the abundances of *Enterobacteriales*, *Lactobacillus*, and *Clostridiales* were influenced by sex in preterm infants. However, in our analysis, *Lactobacillus* and *Clostridiales* were enriched in blood samples collected from women with preterm deliveries after adjusting for infant prenatal sex. Thus, our results suggest that the increased prevalence of *Bacteroides* and *Lactobacillus* in the maternal blood microbiota is associated with PTB.

We acknowledge several limitations of our study. The primary limitation is the small number of patients in the case cohort and the ethnic homogeneity of the participants. Another limitation is the lack of a direct comparison between our blood microbiome data and the oral, gut, and placental microbiome derived from the subjects enrolled in the study. Lastly, although in 2015, obesity rate (body mass index, BMI >30) was 5.3% in Korea (OECD, 2017), our study did not include information on pre-pregnancy BMI. Thus, further studies by other investigators are needed to confirm our results.

In summary, we found that the number and abundances of distinct types of organisms did not change in peripheral blood during healthy pregnancy. However, the microbial diversity in women who experienced PTB differed from that in women delivered at term. While bacterial enrichment did not change during healthy pregnancy, a case-control study demonstrated that several taxa, such as *Bacteroides*, *Lactobacillus*, *Delftia*, and *Pseudomonas* exhibited differential enrichment between women delivered preterm and term. This suggests that changes in the microbiota in various locations in the body during pregnancy can be detected in the blood. Further studies are needed to confirm the composition of the blood microbiota in pregnant women and women with PTB. Moreover, the mechanism by which pathogenic microbes cause infection requires further analysis.

## Author Contributions

Y-AY and YK conceived and designed the experiments. Y-AY, JY, and EK performed the experiments. Y-AY, JY, and EK analyzed the data. YK contributed reagents, materials, and analysis tools. Y-AY and YK wrote the manuscript.

## Conflict of Interest Statement

The authors declare that the research was conducted in the absence of any commercial or financial relationships that could be construed as a potential conflict of interest.

## Footnotes

1 http://qiime.org/home_static/dataFiles.html
